# The impact of metabolic reprogramming in hepatocellular carcinoma on T cell

**DOI:** 10.3389/fimmu.2025.1696113

**Published:** 2025-11-10

**Authors:** Dong-Xu Liao, Xiang An, Gui-Xiang Huang, Tong-Ling Yuan

**Affiliations:** 1 Department of Hepatobiliary and Pancreatic Surgery, Sichuan Provincial People’s Hospital, School of Medicine, University of Electronic Science and Technology of China, Chengdu, Sichuan, China; 2 Department of Emergency Surgery, Sichuan Provincial People’s Hospital, School of Medicine, University of Electronic Science and Technology of China, Chengdu, Sichuan, China; 3 General Practice Center, Sichuan Provincial People’s Hospital, School of Medicine, University of Electronic Science and Technology of China, Chengdu, Sichuan, China

**Keywords:** hepatocellular carcinoma (HCC), metabolic reprogramming, tumor microenvironment (TME), T cell-mediated antitumor immunity, metabolic-immune targeted combination therapies

## Abstract

Hepatocellular carcinoma (HCC) is the predominant type of liver cancer, characterized by high incidence and mortality rates. Despite advancements in surgical and systemic therapies, the prognosis remains poor due to the asymptomatic nature of early-stage HCC. Metabolic reprogramming in HCC cells usually creates an immunosuppressive tumor microenvironment (TME), thereby impeding T cell-mediated antitumor immunity. This review focuses on the metabolic reprogramming patterns in HCC, their impact on T cell function, and the potential of metabolic-immune targeted combination therapies. We emphasize that nutrient competition and the accumulation of inhibitory metabolites are key mechanisms underlying T cell suppression in the TME. This review provides an update on the complex metabolic-immune interactions and helps to identify new therapeutic targets to improve the efficacy of immunotherapy for HCC.

## 1 Introduction

Hepatocellular carcinoma (HCC), one of the main types of liver cancer, accounts for over 90% of all liver cancer cases. Its incidence and mortality rates rank among the highest globally (1, 2). In 2022, there were approximately 866,136 new cases and 758,725 deaths worldwide. The Asia-Pacific region bore the heaviest burden of HCC, accounting for 71% of global new cases and 42% of deaths in this area (3). In recent years, although significant progress has been made in curative treatments such as surgical resection, liver transplantation, and local ablation, as well as systemic therapies like targeted therapy and immunotherapy, the overall prognosis for HCC remains poor (4–7). The core challenge lies in the fact that HCC is often asymptomatic in its early stages. Most patients present with advanced tumors when clinical symptoms become apparent, thereby missing the opportunity for curative surgery (8). Therefore, there is an urgent need to obtain a deeper understanding of the molecular pathogenesis and identify new therapeutic targets in current HCC fields.

For patients with advanced HCC ineligible for surgery, systemic therapy has been considered as the primary approach to prolong survival and improve life quality (9). Over the past decade, multi-kinase inhibitors such as sorafenib and lenvatinib have become the standard first-line treatments. However, their objective response rate (ORR) typically ranges between 10% and 20%, and patients inevitably develop primary or secondary resistance (10). Nowadays, the emergence of immune checkpoint inhibitors (ICIs), represented by Programmed death receptor-1 (PD-1)/Programmed Death-Ligand 1 (PD-L1) inhibitors, has brought revolutionary breakthroughs in the treatment of advanced HCC (11). ICIs can “release the brakes,” reactivating suppressed T cells and restoring their ability to kill tumor cells by blocking the binding of PD-L1 on the surface of tumor cells to PD-1 on the surface of T cells (12). However, clinical practice has shown that the ORR of ICI monotherapy for HCC is only 15%-20% (13). Even with immune combination therapies like the “atezolizumab + bevacizumab” (T+A regimen), which increase the ORR to approximately 30%, most patients still do not respond (primary resistance) or experience disease progression after initial effectiveness (secondary resistance) (14).

Metabolic reprogramming—the adaptive alteration of cellular metabolic patterns by cancer cells to meet their demands for rapid proliferation and survival—is now recognized as one of the hallmark characteristics of cancer (15, 16). This reprogramming enables them to overcome the limitations of normal metabolism, efficiently acquire and utilize limited nutrients (e.g., glucose and glutamine) to sustain their own survival and support uncontrolled rapid proliferation (17, 18). Given the pervasiveness and central role of these metabolic alterations in tumor biology, cellular metabolic reprogramming and its accompanying changes in energy production and utilization have been unequivocally identified as a hallmark feature of cancer (19). Thus, in-depth investigation into the unique and often aberrant metabolic mechanisms of tumor cells—such as their high dependence on aerobic glycolysis (the Warburg effect) and glutaminolysis—is widely regarded as an extremely promising research field with significant scientific value and clinical application potential (20, 21). Exploring metabolic reprogramming in HCC helps reveal its fundamental biological behavior and opens entirely new avenues for developing novel diagnostic biomarkers, prognostic assessment tools, and therapeutic strategies (22, 23).

The therapeutic bottlenecks of ICIs have profoundly highlighted significant gaps in our understanding of HCC immune evasion mechanisms. Tumors do not simply evade immune surveillance by expressing checkpoint molecules like PD-L1; instead, this evasion is underpinned by a more complex and robust inhibitory network (24–26). At the core of this network lies the “soil” upon which tumors thrive and develop—the tumor microenvironment (TME) (27–29). As an active digestive organ, metabolic changes in HCC profoundly impact the antitumor activity of the immune system within the TME (23, 30, 31). Therefore, it is of great significance to investigate the intrinsic mechanisms leading to ICI treatment resistance, particularly the immunosuppressive mechanisms caused by metabolic alterations in the TME, and identifying new targets and strategies to overcome resistance and sensitize immunotherapy in the field of liver cancer research.

## 2 Metabolic reprogramming patterns in HCC cells

Metabolic reprogramming has been recognized as one of the ten core hallmarks of cancer (15). This is not merely a passive consequence of malignant proliferation in tumor cells, but a key driver enabling them to actively adapt to the microenvironment and sustain survival, invasion, and metastasis. Compared to normal hepatocytes, HCC cells have undergo significant metabolic alterations to support their biological behaviors of unlimited proliferation, invasion, and metastasis (32). Recent proteomic and metabolomic studies have revealed the remodeling of multiple core metabolic pathways in HCC (33–35).

### 2.1 Glucose metabolic reprogramming: the Warburg effect and beyond

#### 2.1.1 Glycolysis

It is well-known that enhanced glycolysis is the most classic and extensively studied metabolic feature of HCC cells, known as the “Warburg Effect” (36–38). Cancer cells preferentially convert glucose into lactate even under oxygen-sufficient conditions, rather than fully oxidizing it via mitochondrial tricarboxylic acid (TCA) cycle and oxidative phosphorylation (OXPHOS) (39, 40). This shift is driven by the upregulation of key enzymes and transporters, including glucose transporter 1 (GLUT1) (41), hexokinase 2 (HK2) (42), pyruvate kinase M2 isoform (PKM2) (43), and lactate dehydrogenase A (LDHA) (44). Hexokinase 2 (HK2) enhances glucose uptake by binding to the mitochondrial voltage-dependent anion channel (VDAC) (45, 46). Its inhibitor, Chrysin, can induce cancer cell apoptosis (47). Conversely, high expression of the pyruvate kinase M2 isoform (PKM2) promotes lactate production (48). The natural compound Shikonin effectively blocks glycolysis by inhibiting PKM2 and sensitizes cancer cells to sorafenib (49, 50). Hypoxia-inducible factor HIF-1α plays a central regulatory role in this process. It drives the expression of enzymes such as HK2 and aldolase A (ALDOA) (51, 52). Additionally, metformin can inhibit glycolytic flux by activating the AMPK pathway to inhibit phosphofructokinase 1 (PFK1) (53, 54).

This seemingly inefficient energy production strategy confers the following survival advantages to cancer cells:

1. Rapid energy supply: Accelerated glycolysis generates ATP at a significantly faster rate compared to OXPHOS, meeting the high energy demands of rapidly proliferating cancer cells (55).
2. Biosynthetic precursor provision: Glycolytic intermediates—such as glyceraldehyde-3-phosphate and phosphopentoses—are diverted into the pentose phosphate pathway (PPP) and serine synthesis pathway. These supply precursors for nucleic acids, non-essential amino acids, and reducing equivalents, supporting macromolecular biosynthesis and redox homeostasis (56, 57).
3. Microenvironment acidification: Massive lactate efflux acidifies the TME. This suppresses immune cell activity to promote immune evasion while enhances tumor cell invasiveness and metastatic potential (58–60). Notably, lactate, the end product of glycolysis, is not merely a metabolic waste but can directly regulate gene expression through histone lactylation (such as the H3K18la modification), thereby influencing inflammatory responses within the TME (61).

#### 2.1.2 Pentose phosphate pathway

Pentose Phosphate Pathway (PPP), which is co-activated in HCC, serves not only as an alternative energy source but also as a core strategy for maintaining redox homeostasis and biosynthesis (62). Glucose-6-phosphate dehydrogenase (G6PD), the rate-limiting enzyme of PPP, drives malignant progression through two key pathways via its overexpression:

Antioxidant Defense: Catalyzes the generation of NADPH to reduce glutathione (GSH), thereby neutralizing reactive oxygen species (ROS) and protecting tumor cells from oxidative damage (63).

Pro-Metastatic Mechanism: Activates STAT3 phosphorylation, inducing the expression of key epithelial-mesenchymal transition (EMT) molecules, promoting invasion and metastasis (64).

Notably, transketolase (TKT) exhibits tissue-specific overexpression in HCC (65). Its abnormal elevation depletes reduced NADP^+^, leading to ROS accumulation, which subsequently activates pro-survival signals (66, 67). Targeted intervention confirmed: the TKT inhibitor Oxythiamine significantly increases intracellular ROS levels (68). When combined with Sorafenib, it achieved over 60% tumor growth inhibition in a patient-derived xenograft (PDX) model of 292 HCC patients (69). The biological significance of PPP extends beyond antioxidation – the ribose-5-phosphate it generates – provides essential precursors for nucleotide synthesis, directly supplying the DNA/RNA raw materials for rapid tumor cell proliferation (70, 71).

#### 2.1.3 Gluconeogenesis

In sharp contrast, gluconeogenesis is systematically inhibited in HCC (72, 73). Fructose-1,6-bisphosphatase 1 (FBP1) is a key tumor-suppressing enzyme that is inactivated through multi-layered regulation: 1) Epigenetic Silencing: Histone deacetylase-mediated loss of H3K27ac modification suppresses FBP1 promoter activity (ChIP-seq confirmed enrichment of HDAC1/2 at the FBP1 promoter region) (74); and 2) Ubiquitination-Dependent Degradation: The E3 ubiquitin ligase TRIM28 directly binds FBP1, promoting its 26S proteasome-dependent degradation (validated by co-immunoprecipitation assays) (75). FBP1 deficiency releases the inhibition on the glycolytic rate-limiting enzyme PFK1, causing a surge in glycolytic flux (76, 77).

Another key enzyme, phosphoenolpyruvate carboxykinase (PCK1), exhibits a “dual nature”: Under physiological conditions, PCK1 maintains metabolic homeostasis by inhibiting TCA cycle intermediate efflux (cataplerosis) (78).

In HCC, AKT-mediated phosphorylation at Ser90 triggers its aberrant translocation to the endoplasmic reticulum. At the same site, it phosphorylates INSIG1/2 proteins, releases the lipid synthesis transcription factor SREBP1, and unexpectedly drives lipogenesis and tumor progression (79).

Rescue strategies targeting suppressed gluconeogenesis show:

The histone deacetylase inhibitor SAHA upregulates FBP1 expression by 2.5-fold, while lowering he glycolysis rate in HepG2 cells by 40% (74).

The glucocorticoid Dexamethasone restores PCK1/FBP1 expression by activating the transcriptional coactivator PGC-1α, thereby reversing the Warburg effect and reducing tumor volume by 52% in animal models (80, 81).

#### 2.1.4 Tricarboxylic acid cycle

Remodeling of the Tricarboxylic Acid (TCA) Cycle plays a pivotal role in HCC metabolic adaptation (82). The anaplerotic reaction driven by pyruvate carboxylase (PC) replenishes TCA intermediates by generating oxaloacetate (83). Treatment of HepG2 cells with its small molecule inhibitor UK-5099 reduced aspartate synthesis by 70%, causing nucleotide synthesis impairment and cell cycle arrest (84). Conversely, downregulation of isocitrate dehydrogenase 2 (IDH2) promotes extracellular matrix degradation and metastasis by negatively regulating matrix metalloproteinase MMP9 (immunohistochemistry shows a significant negative correlation between IDH2 and MMP9 expression) (85, 86). For the IDH-mutant subtype (mIDH), in a Phase I clinical trial, the oral inhibitor Ivosidenib (AG-120) extended progression-free survival by 3.1 months in advanced HCC patients (NCT02073994) (87, 88).

Furthermore, overexpression of malic enzyme 1 (ME1) drives metastasis through a dual pathway: 1) catalyzing malate decarboxylation to generate NADPH, maintaining a reductive microenvironment (89, 90); and 2) the activation of the ROS-ZEB1 signaling axis induces EMT and suggests its potential as a pan-cancer therapeutic target, as evidenced by an 80% decrease in HCC cell invasiveness following ME1 knockdown with shRNA (91).

#### 2.1.5 Glycolytic metabolites and the epigenetic landscape in hepatocellular carcinoma

Metabolic reprogramming directly modulates the epigenetic landscape of both HCC cells and T cells by altering the abundance of key metabolites. Acetyl-CoA, derived from glucose and lipid metabolism, serves as the primary donor for histone acetylation. Its accumulation in HCC increases histone acetylation levels, thereby activating genes associated with cell proliferation. S-adenosylmethionine (SAM), produced via the methionine cycle, acts as the principal methyl donor for histone methylation. Increased SAM generation promotes histone methylation, leading to the activation of genes involved in tumor progression (92, 93). Alpha-ketoglutarate (α-KG), an intermediate of the TCA cycle, is an essential substrate for histone and DNA demethylation. Its reduction inhibits demethylation processes, thereby promoting tumorigenesis (94, 95).

Furthermore, metabolic reprogramming influences the epigenetic state of T cells. For instance, elevated lactate levels induce histone lactylation (H3K18la) within T cells, which suppresses T-cell activation (96). Future research should focus on elucidating the intricate coupling mechanisms between metabolism and epigenetics. Developing novel therapeutic strategies that target metabolic pathways to restore metabolite balance, reverse epigenetic alterations, inhibit tumor progression, and enhance the efficacy of immunotherapy represents a promising direction.

### 2.2 Lipid metabolic reprogramming: the *de novo* synthesized “fuel depot” and signaling hub

Lipid metabolism, including synthesis, storage, and degradation of lipids, is crucial for cellular function and membrane integrity, and its dysregulation is associated with diseases like cancer (97, 98).

#### 2.2.1 Fatty acid uptake

Liver is the central organ of lipid metabolism, and the abnormalities in lipid metabolism in HCC involve multiple aspects (99, 100). In terms of fatty acid uptake and transport, transmembrane transporters such as CD36 and fatty acid transport proteins (FATPs) are upregulated in HCC tissues, promoting fatty acid uptake (101, 102). Meanwhile, the upregulation of CD36 can induce EMT, thereby promoting invasion and metastasis (103, 104). Among fatty acid-binding proteins (FABPs), FABP1 and FABP5 are highly expressed in HCC (105, 106). FABP1 promotes cell migration, angiogenesis, and metastasis through the VEGFR2/SRC and FAK/CDC42 signaling pathways, while FABP5 promotes lipid accumulation and cell proliferation by activating the HIF-1α axis. This is associated with poor prognosis (107, 108). Lipoprotein lipase (LPL) hydrolyzes triglycerides (TAGs) into free fatty acids (FFAs), promoting the uptake of lipoproteins by cells (109–111). Its expression is higher in advanced HCC (stages III/IV), and inhibiting LPL can hinder the proliferation of HCC cells (112).

#### 2.2.2 *De novo* lipogenesis

Regarding *de novo* lipogenesis (DNL), which is abnormally activated in HCC and serves as a hallmark feature, provides membrane components and signaling molecules for rapidly proliferating tumor cells (113–115). The expression of key enzymes is significantly increased. For example, ATP citrate lyase (ACLY) cleaves citrate exported from the mitochondria into cytoplasmic acetyl-CoA (a substrate for fatty acid synthesis) (116, 117). Oncogenic drivers such as PI3K/AKT activate ACLY and the Warburg effect through feedback loops (107, 118). Acetyl-CoA carboxylase (ACC) is the rate-limiting enzyme for fatty acid synthesis and catalyzes the formation of malonyl-CoA from acetyl-CoA (119, 120). Its liver-specific inhibitor ND-654 can inhibit hepatic DNL, inflammation, and HCC development (121). Fatty acid synthase (FASN) synthesizes fatty acids such as palmitate from malonyl-CoA and acetyl-CoA (122). Its overexpression indicates a poor prognosis, and inhibiting FASN significantly suppresses the growth and tumorigenesis of HCC cells. Targeting FASN and related *de novo* lipogenesis is a potential therapeutic strategy (123, 124). Stearoyl-CoA desaturase 1 (SCD1) converts saturated fatty acids into monounsaturated fatty acids (MUFAs) (125). Interfering with SCD1 effectively inhibits HCC progression, while its overexpression is associated with shorter disease-free survival. SCD1 exerts its effects by regulating p53, WNT/β-catenin, EGFR, and autophagy. Inhibiting SCD1 can induce endoplasmic reticulum (ER) stress, which differentiates liver cancer stem cells and thereby sensitizes them to sorafenib (107, 126–128). Acyl-CoA synthase long-chain family member 4 (ACSL4) prefers arachidonic acid, and its overexpression stabilizes c-MYC through the ERK/FBW7/c-MYC axis, promoting tumor formation *in vivo* and *in vitro*. It is a potential prognostic marker and therapeutic target (129, 130).

In terms of transcriptional regulation, sterol regulatory element-binding protein 1 (SREBP1) is the core transcriptional activator of *de novo* lipogenesis (DNL) and is significantly more highly expressed in HCC tumor tissues than in adjacent non-tumor tissues (131–133). Downregulating SREBP1 inhibits the proliferation, migration, and invasion of HCC cells and induces apoptosis and SREBP1 is a prognostic marker for HCC (132). Liver X receptor (LXR), a member of the nuclear receptor superfamily, regulates cholesterol homeostasis and *de novo* lipogenesis by transactivating SREBP1, FASN, and others (134). LXRα is downregulated in HCC tissues (135–137). LXR agonist T0901317 can upregulate LXRα while downregulate GLUT1 and MMP9, thereby inhibiting HCC progression (136). Nevertheless, long-term use of LXR agonists combined with oxidative stress and a high-fat diet can induce a non-alcoholic steatohepatitis (NASH)-like condition in mice and progress to HCC, demonstrating the complexity of the effects (138).

#### 2.2.3 Fatty acid oxidation

Regarding the dysregulation of fatty acid β-oxidation (FAO), despite the frequent lack of nutrients in tumor centers due to inadequate vascularization, FAO remains an important catabolic pathway for providing energy and anabolic precursors for cell growth (139). Carnitine palmitoyltransferase 1 (CPT1) is the rate-limiting enzyme for FAO and is located on the outer mitochondrial membrane. With abundant glucose, ACC forms a complex with CPT1A to prevent its localization to the mitochondria. During glucose deprivation, the complex dissociates, and free CPT1A localizes to the mitochondrial membrane to enhance FAO (140–142). ACC and CPT1A together protect HCC cells against metabolic stress (143, 144). Acyl-CoA dehydrogenases, including medium-chain (MCAD) and long-chain (LCAD) acyl-CoA dehydrogenases, catalyze the first step of mitochondrial FAO (145–147). They are downregulated by hypoxic stress, inhibiting fatty acid breakdown and promoting HCC cell proliferation. Forced expression of these enzymes can decrease the lipid accumulation caused by hypoxia (148–150). However, knocking down LCAD can promote tumor growth *in vivo* by inhibiting the Hippo pathway (151). Lipolysis provides the fatty acid substrates for FAO (152). Monoglyceride lipase (MAGL) catalyzes the conversion of monoglycerides into FFAs and glycerol (153, 154). Its high expression in cancer promotes HCC cell proliferation and invasion by generating signaling lipids (including monoglycerides and FFAs), while the NF-κB signaling pathway is involved in MAGL-mediated EMT (155). Adipose triglyceride lipase (ATGL) initiates the hydrolysis of TAGs into diacylglycerols (DAGs) and FFAs (156, 157). High expression of ATGL in HCC tissues leads to elevated levels of DAGs and FFAs, which are associated with poor prognosis. lncRNA-NEAT1 induces abnormal lipolysis in HCC cells by upregulating ATGL, driving cell growth (158).

#### 2.2.4 Cholesterol synthesis

Regarding cholesterol metabolism disorders, dysregulation of cholesterol biosynthesis is a common event in HCC. During the hepatocarcinogenesis, there is new biochemical crosstalk between *de novo* lipogenesis and the cholesterol biosynthesis pathway (159). HMG-CoA reductase (HMGCR), the rate-limiting enzyme in the mevalonate pathway of cholesterol synthesis, can be inhibited by statins (160–162). For human HCC samples, the upregulation of HMGCR is accompanied by increased mitochondrial cholesterol levels. Inhibiting HMGCR or squalene synthase (the enzyme in the first step of cholesterol biosynthesis) to deplete cholesterol can enhance the sensitivity of HCC cells to chemotherapy (107, 163, 164). Squalene synthase inhibitors (e.g., YM-53601) can synergistically mediate HCC growth arrest and cell death with doxorubicin *in vivo* (163).

Regarding other related pathways, peroxisome proliferator-activated receptor α (PPARα) regulates the constitutive transcription of genes related to fatty acid transport and TAG homeostasis (165). Long-term administration of PPARα ligands accelerates hepatocyte proliferation, increases ROS production, and leads to HCC in rodents (166–168). The oncogene MYC directly acts as a transcriptional amplifier for specific PPARα target genes (such as KRT23, which promotes hepatocyte proliferation and potential HCC) (169). Cytochrome P450 family 4 (CYP4) is a group of ω-hydroxylases involved in fatty acid transformation. Lowered expression of CYP4 is associated with liver fat accumulation and the pathogenesis of NASH. CYP4Z1 and its pseudogene CYP4Z2P are highly expressed in breast cancer and promote angiogenesis, but their roles in fatty acid metabolism in HCC remain unclear (170, 171).

### 2.3 Amino acid metabolic reprogramming: glutamine as the central hub

The progression of HCC is accompanied by profound metabolic reprogramming, in which the dysregulation of amino acid metabolism plays a key role in meeting the vigorous biosynthetic demands of tumor cells, maintaining redox homeostasis, and evading immune surveillance (172, 173).

As the central organ of amino acid metabolism, the liver undergoes extensive changes in amino acid uptake, synthesis, degradation, and waste disposal pathways during carcinogenesis, providing an important window into understanding the pathogenesis of HCC and developing targeted therapies (174). Glutamine is an important nitrogen and carbon source for HCC cells, and its abnormal metabolism (glutaminolysis) is a significant characteristic of HCC (175–177). Glutaminase (GLS) is the rate-limiting enzyme for glutaminolysis, with two subtypes playing distinct roles in HCC (178, 179). GLS1 (kidney-type) is universally overexpressed in HCC, promoting cell proliferation and colony formation by activating the AKT/GSK3β/Cyclin D1 axis (180). Its upregulation is associated with advanced clinical pathological features and a stem cell phenotype, regulating cancer stem cell properties through the ROS/WNT/β-catenin pathway (181). Knocking out GLS1 can inhibit tumorigenicity *in vivo*, suggesting it is an important target for eradicating cancer stem cells (182). GLS2 (liver-type) typically exerts its tumor - suppressing function by promoting ferroptosis, and its regulatory role in glutaminolysis is essential for tumor growth inhibition (183–185). Additionally, GLS2 inhibits EMT through the Dicer-miR-34a-Snail axis, exerting its non-glutamine catabolism function to suppress migration and invasion of HCC cells (186).

To meet the dramatically increased demand for amino acids, HCC cells heavily rely on transmembrane transporters to uptake amino acids from the microenvironment, making key transporters potential therapeutic targets (187, 188). SLC1A5/ASCT2 is the primary Na^+^-dependent glutamine transporter, whose expression is significantly higher in HCC tumor tissues compared to adjacent non-tumor tissues, and positively correlated with tumor size, making it a potential prognostic indicator (189). SLC7A5/LAT1 forms a heterodimer with 4F2hc (SLC3A2/CD98) to transport large neutral amino acids (such as leucine) (190). LAT1 expression is significantly elevated in HCC lesions and is associated with tumor growth (191, 192). Hippo pathway effectors YAP/TAZ increase amino acid uptake by upregulating LAT1 expression, activating mTORC1, and promoting proliferation (193). Patients with high LAT1 expression have significantly shorter survival rates. Its structural and functional mechanisms are relatively clear, making it a highly promising therapeutic target. The selective inhibitor JPH203 showed partial response or stable disease in a Phase I trial for advanced solid tumors (including cholangiocarcinoma) and is currently in Phase II trials (for cholangiocarcinoma). Another inhibitor, QBS10072S, is undergoing a Phase I trial (including HCC patients) (181, 194).

Glutamic Oxaloacetic Transaminase 1 (GOT1) coordinates glucose and amino acid metabolism to meet nutritional demands (195). Oncogenic KRAS mutations have been found to depend on GOT1 to support long-term cell proliferation (196). lncRNA TMPO-AS1 accelerates HCC progression by targeting the miR-429/GOT1 axis (197). Abnormalities in ornithine cycle (urea cycle) enzymes are common in HCC (197). Carbamoyl Phosphate Synthetase 1 (CPS1), the rate-limiting enzyme in the first step of the urea cycle, is a hypermethylated gene in HCC and is downregulated by aflatoxin B1, thereby inhibiting proliferation and inducing apoptosis (198–201). Argininosuccinate Synthase 1 (ASS1) is another rate-limiting enzyme that is frequently deficient in tumors. ASS1 deficiency in tumors is both a prognostic biomarker and a predictor of sensitivity to arginine-deprivation therapy (202). Due to DNA methylation, ASS1 expression is significantly reduced in HCC patients, and stable silencing of ASS1 promotes migration and invasion (203, 204). ASS1 deficiency increases STAT3 Ser727 phosphorylation and promotes metastasis by upregulating the inhibitor of differentiation 1 (205). ASS1 inhibits HCC metastasis and is a potential diagnostic and therapeutic target (202). Arginase-1 (ARG1) is expressed in the cytoplasm of hepatocytes and is involved in anti-inflammatory, tumor immune, and immunosuppressive functions. Overexpression of ARG1 enhances the viability, migration, and invasion of HCC cells and significantly increases the expression of key EMT factors (vimentin, N-cadherin, β-catenin) (206). Ornithine Transcarbamylase (OTC) is located in the mitochondrial matrix. OTC deficiency disrupts the urea metabolic pathway, leading to elevated blood ammonia levels (potentially life-threatening in severe cases). OTC silencing promotes HCC cell proliferation, and clinical data show that patients with low OTC levels have shorter overall survival (207–210).

**Table 1** summarizes the key metabolic pathways, molecules, targeted inhibitors, and therapeutic significance in HCC. It serves as a generalization and supplement to the detailed metabolic reprogramming patterns described earlier. This allows readers to quickly review and compare these key pieces of information, and provides a reference for the subsequent discussion on the impact of metabolic reprogramming on T cells and therapeutic strategies.

**Table 1:** Key pathways and targets of metabolic reprogramming in HCC.

Metabolic type	Key molecules/pathways	Function/impact	Targeted inhibitors	Therapeutic significance	Refs
Glucose Metabolism	GLUT1/HK2/PKM2/LDHA	Enhanced glycolysis → Lactate accumulation → TME acidification	Shikonin (PKM2 inhibitor)	Sensitizes cancer cells to sorafenib	(39, 47, 48)
	PPP pathway (G6PD/TKT)	Provides nucleic acid precursors → Promotes metastasis	Oxythiamine (TKT inhibitor)	60% tumor growth inhibition in PDX models	(60, 67)
Lipid Metabolism	SREBP1/FASN/ACC/SCD1	*De novo* lipogenesis → Membrane components/signaling molecules	TVB-3664 (FASN inhibitor)	Stabilizes MHC-I → Synergizes with anti-PD-L1	(116, 211)
	HMGCR	Cholesterol synthesis ↑ → Chemoresistance	Statins	Enhances chemotherapy sensitivity	(156)
Amino Acid Metabolism	SLC1A5 (ASCT2)/SLC7A5	Glutamine/leucine uptake ↑ → mTORC1 activation	CB-839 (GLS inhibitor)	Alleviates T cell nutrient deprivation	(182, 212)
	IDO1/TDO2	Tryptophan → Kynurenine → Treg differentiation ↑	Epacadostat (IDO1 inhibitor)	Reverses immunosuppression	(213, 214)

## 3 The alteration of key metabolites in the TME of HCC

TME is a complex ecosystem composed of tumor cells, immune cells, stromal cells, blood vessels, and various cytokines and chemicals (215). Within this ecosystem, tumor cells are not passively present but actively reshape the environment to facilitate their own survival through various means (216). Metabolic reprogramming is one of their most powerful tools for this transformation (217). By altering the uptake, utilization of key nutrients, and secretion of metabolic byproducts, tumor cells can launch a “metabolic war” against the immune system, ultimately establishing an immunosuppressive microenvironment (218).

### 3.1 Competitive nutrient deprivation

The rapid, uncontrolled proliferation of tumor cells is one of the core biological characteristics (15). This process creates an extremely high dependence on nutrients within the microenvironment. To win this fierce competition for survival, HCC cells have evolved a series of complex metabolic reprogramming strategies. The core of these strategies lies in effectively plundering core resources such as glucose and amino acids from the TME by upregulating the expression of key nutrient transporters on the cell membrane (217, 219). This meets their biosynthetic and energy metabolism demands while causing local nutrient deprivation, thereby suppressing the function of competitors like immune cells (29).

#### 3.1.1 Glucose

“Warburg effect” is one of the most prominent metabolic features of HCC. However, this seemingly inefficient metabolic pattern provides cancer cells with rapid ATP energy and abundant biosynthetic precursors (such as ribose, glycerol, etc.) to support their rapid proliferation (220, 221).

##### 3.1.1.1 Upregulation of glucose transporters

To meet the enormous demand for glucose, HCC cells significantly upregulate the expression of glucose transporters (GLUTs). As the most extensively studied transporter, GLUT1 is highly expressed in the majority of HCC tissues. Its expression level is closely correlated with tumor aggressiveness, glycolytic flux, and poor patient prognosis (222). A study has confirmed that GLUT1 mRNA and protein levels are significantly higher in HCC cells compared to adjacent non-tumor tissues. Knocking down GLUT1 effectively inhibits HCC cell growth and migration (222). GLUT2, also responsible for glucose transport in normal hepatocytes, is upregulated in HCC and associated with poor prognosis (223, 224). GLUT3 has also been shown to be upregulated in HCC, and promote glucose uptake and driving glycolytic reprogramming alongside other GLUTs (225).

The upregulation of GLUTs is regulated by complex signaling networks, which are often abnormally activated in HCC:

PI3K/AKT/mTOR Pathway: This is one of the core signaling pathways in cancer, comprehensively regulating cell growth, proliferation, and metabolism. Its abnormal activation is a major driver of altered glucose metabolism in tumors (226).

HIF-1α: Hypoxia-inducible factor-1α (HIF-1α) is activated under the hypoxic conditions common in the TME. As a key transcription factor, it can directly upregulate the expression of GLUT1, and various glycolytic enzymes, thereby enhancing cell survival capability in hypoxic environments (227, 228).

FOXM1: The transcription factor FOXM1 plays a significant oncogenic role in HCC. FOXM1 can transcriptionally activate GLUT1 expression by directly binding to its promoter region, systematically regulating glycolysis in HCC and promoting tumorigenesis (229).

#### 3.1.2 Glutamine and glutamate

If glucose is the primary fuel for HCC cells, glutamine is their indispensable “secondary fuel” and a key nitrogen source (176). Glutaminolysis provides HCC cells with energy, biosynthetic precursors, and key substances for maintaining redox homeostasis (230).

HCC cells voraciously uptake glutamine and glutamate by upregulating various amino acid transporters, leading to glutamine and glutamate deficiency in the TME (176).

SLC1A5 (ASCT2) is the primary and most extensively studied glutamine transporter. For HCC, SLC1A5 expression is widely upregulated, and its high expression is significantly correlated with tumor growth, metastasis, and poor patient prognosis. Targeted inhibition of SLC1A5 effectively suppresses HCC growth (189, 231).

SLC38A1 (SNAT1) and SLC38A2 (SNAT2): According to research data, these two transporters are significantly upregulated in HCC. Notably, SLC38A2 is even the highest expressed glutamine transporter in certain HCC models. They work synergistically with SLC1A5 to ensure sufficient glutamine supply (232–234).

SLC1A3 (EAAT1/GLAST): As a high-affinity glutamate transporter, SLC1A3 expression in HCC tissues is significantly higher than in normal tissues. Its high expression is closely associated with worse tumor grade, pathological stage, overall survival, and immune evasion phenomena, making it a potential novel prognostic biomarker and therapeutic target (235).

#### 3.1.3 Methionine

Hoffman effect refers to the dependence of cancer cells on methionine. All types of cancer cells exhibit an “addiction” to methionine (236). This phenomenon arises from excessive transmethylation in cancer cells, leading to a greater requirement for methionine compared to normal cells (237). HCC cells commonly exhibit an imbalance in the expression of the methionine adenosyltransferase (MAT) family, characterized by downregulation of the tumor-suppressive MAT1A and upregulation of the oncogenic MAT2A, resulting in abnormalities in the S-adenosylmethionine (SAM) synthesis pathway (238, 239). In diverse human tumor cell lines, ASS1 (argininosuccinate synthase 1) expression is lost, preventing the tumor from regenerating methionine via homocysteine, thereby creating a “methionine auxotrophic” phenotype (240, 241). HCC cells highly express the methionine transporter SLC43A2, and its expression level positively correlates with tumor malignancy (242). This ultimately leads to methionine deficiency in the TME.

#### 3.1.4 Cystine/cysteine

Cystine/cysteine serves as a nutrient supporting HCC growth and participates in the intracellular redox balance of tumor cells (243). Oxidative stress is an inevitable consequence of the rapid metabolism and proliferation of cancer cells, and ferroptosis—an iron-dependent, lipid peroxidation-driven form of programmed cell death—poses a serious threat to cancer cells. HCC cells can establish a robust defense system to resist ferroptosis by reprogramming cysteine metabolism (244).

The core strategy for HCC cells to resist ferroptosis is the upregulation of the cystine/glutamate antiporter, System Xc^−^. This system consists of two subunits, SLC7A11 and SLC3A2, with SLC7A11 being the core catalytic subunit responsible for its transport function (243). SLC7A11 is abnormally highly expressed in HCC. It transports extracellular cystine (the disulfide-bonded form of two cysteine molecules) into the cell while pumping intracellular glutamate out. This consequently causes cystine deficiency in the TME (245, 246).

#### 3.1.5 Arginine

Arginine plays a complex dual role in tumor biology. It is a precursor for protein synthesis, polyamines, and nitric oxide (NO) generation, and functions as an important signaling molecule (247). The reprogramming of arginine metabolism in HCC cells reflects their high adaptability (23).

A particularly notable phenomenon is the loss or downregulation of expression of argininosuccinate synthase 1 (ASS1), the key rate-limiting enzyme in the arginine synthesis pathway in many HCC cells. This renders these HCC cells “arginine auxotrophs,” dependent on acquiring arginine from the external environment to survive (213, 248). To compensate for this deficiency, HCC cells upregulate cationic amino acid transporters (CATs), such as SLC7A1 (CAT1) and SLC7A2 (CAT2), to enhance arginine uptake, causing arginine deficiency in the TME (249). Simultaneously, tumor cells downregulate enzymes that break down arginine into polyamines (e.g., ARG1) to accumulate high intracellular levels of arginine (206, 250).

#### 3.1.6 Tryptophan

Tumors development requires nutrients and need to evade immune system surveillance and attack. HCC cells exploit tryptophan metabolism to create an immunosuppressive microenvironment through metabolic reprogramming (251, 252).

HCC cells increase tryptophan uptake by upregulating transporters like the L-type amino acid transporter 1 (LAT1/SLC7A5) (193, 253). HCC cells and other cells within their microenvironment (e.g., immune cells) highly express the two key rate-limiting enzymes in the tryptophan catabolic pathway—the kynurenine pathway: indoleamine 2,3-dioxygenase 1 (IDO1) and tryptophan 2,3-dioxygenase 2 (TDO2). IDO1/TDO2 are upregulated in various cancers and associated with poor prognosis (254). In HCC, IDO1 overexpression is closely linked to malignant tumor behavior and an immunosuppressive state (255, 256). Concurrently, the activity of IDO1/TDO2 depletes tryptophan in the TME.

### 3.2 Oncometabolites

The development and progression of HCC are accompanied by profound cellular metabolic reprogramming. To support their unlimited proliferation, cancer cells preferentially adopt a metabolic pathway that involves glycolysis even in the presence of oxygen, which significantly enhances the efficiency of glucose uptake and utilization. Traditionally, it has been believed that the main consequence of this metabolic pattern is the competition for key nutrients such as glucose with immune cells, leading to “malnutrition” and impaired function of immune cells (257, 258). This is considered a relatively passive form of suppression. However, this perspective overlooks the “output end” of the metabolic processes. In addition to passively consuming nutrients, HCC cells actively secrete metabolites produced during their abnormal metabolic processes, known as “oncometabolites.” These metabolites are “metabolic waste” and biologically active signaling molecules that can act as chemical weapons to directly or indirectly inhibit immune cell functions, reshape the immune microenvironment, and establish a “protective” barrier for tumor growth, invasion, and immune evasion (214, 259). Below, we use the examples of lactate and dog urine acid to illustrate this point.

#### 3.2.1 Lactate

Among all the tumor metabolites, lactate is perhaps the most extensively studied and has the most far-reaching impact. Enhanced aerobic glycolysis metabolism in HCC cells is a key metabolic basis for their proliferation, invasion, and migration, and directly cause the sharp increase in lactate concentration in the TME (260). The large amount of lactate produced within cancer cells needs to be efficiently expelled to maintain a relatively stable intracellular pH and sustain high-throughput glycolysis. This process is primarily mediated by members of the monocarboxylate transporter (MCT) family (261). MCT4 is the main lactate efflux pump, with low affinity for lactate but strong transport capacity, making it particularly suitable for expelling large amounts of lactate from highly glycolytic cells. The expression of MCT4 in HCC tissues is significantly higher than that in adjacent normal liver tissues and is positively correlated with tumor size (262). In HCC, upregulated MCT4 is responsible for pumping lactate produced by cancer cells into the TME, making it a key driver of TME acidification. As a direct product of aerobic glycolysis, lactate concentration in HCC tissues can reach 10–30 mM, far exceeding the 1–2 mM level in normal tissues (263).

#### 3.2.2 Kynurenine

Tryptophan is an essential amino acid in the human body, serving as a key substrate for protein synthesis and as an important signaling molecule regulating immune homeostasis (264). In the TME of HCC, cancer cells systemically activate the tryptophan catabolic pathway by abnormally overexpressing two rate-limiting enzymes, IDO1 and TDO2 (255, 265, 266). These enzymes catalyze the conversion of tryptophan to N-formylkynurenine, triggering a dual pathological effect: local tryptophan depletion and accumulation of the toxic metabolite kynurenine.

#### 3.2.3 Lipid

For HCC, the abnormal and persistent activation of the SREBP pathway leads to a significant increase in *de novo* lipid synthesis (267). After activation, SREBP triggers the expression of a series of downstream effector enzymes that collectively complete the process of fatty acid synthesis, such as fatty acid synthase (FASN), acetyl-CoA carboxylase (ACC), and stearoyl-CoA desaturase (SCD) (268). Meanwhile, the upregulation of HMGCR in HCC results in increased cholesterol synthesis (269). These lipid metabolic disorders in HCC lead to the accumulation of lipid components such as free fatty acids and cholesterol in the TME.

### 3.3 Perspectives on spatial metabolomics

The spatial heterogeneity of TME is a critical factor influencing tumor progression and immune responses. Metabolic states vary significantly across different regions of the TME. For example, areas near blood vessels are typically rich in oxygen and nutrients, whereas regions distant from vessels form hypoxic, acidic, and nutrient-deprived “metabolic deserts.” These distinct metabolic niches recruit and shape functionally diverse T-cell subsets (270–272).

Spatial metabolomics, as an emerging technology, enables precise dissection of the spatial distribution of metabolites within tumor tissues, revealing dynamic metabolic changes across different microenvironments. Understanding this spatial distribution is essential for deciphering immunosuppressive mechanisms, as different metabolic niches may influence T-cell functional states through specific metabolites. For instance, lactate accumulation in hypoxic regions may suppress T-cell activity, while nutrient-rich areas may support T-cell proliferation and function (273, 274).

Therefore, the development of region-specific therapeutic strategies must account for this spatial heterogeneity to achieve more precise treatment outcomes. The application of spatial metabolomics will provide new perspectives for understanding TME complexity and offer important insights for developing personalized treatment strategies.

## 4 Impacts of metabolic reprogramming on T cells in HCC

**Figure 1:**
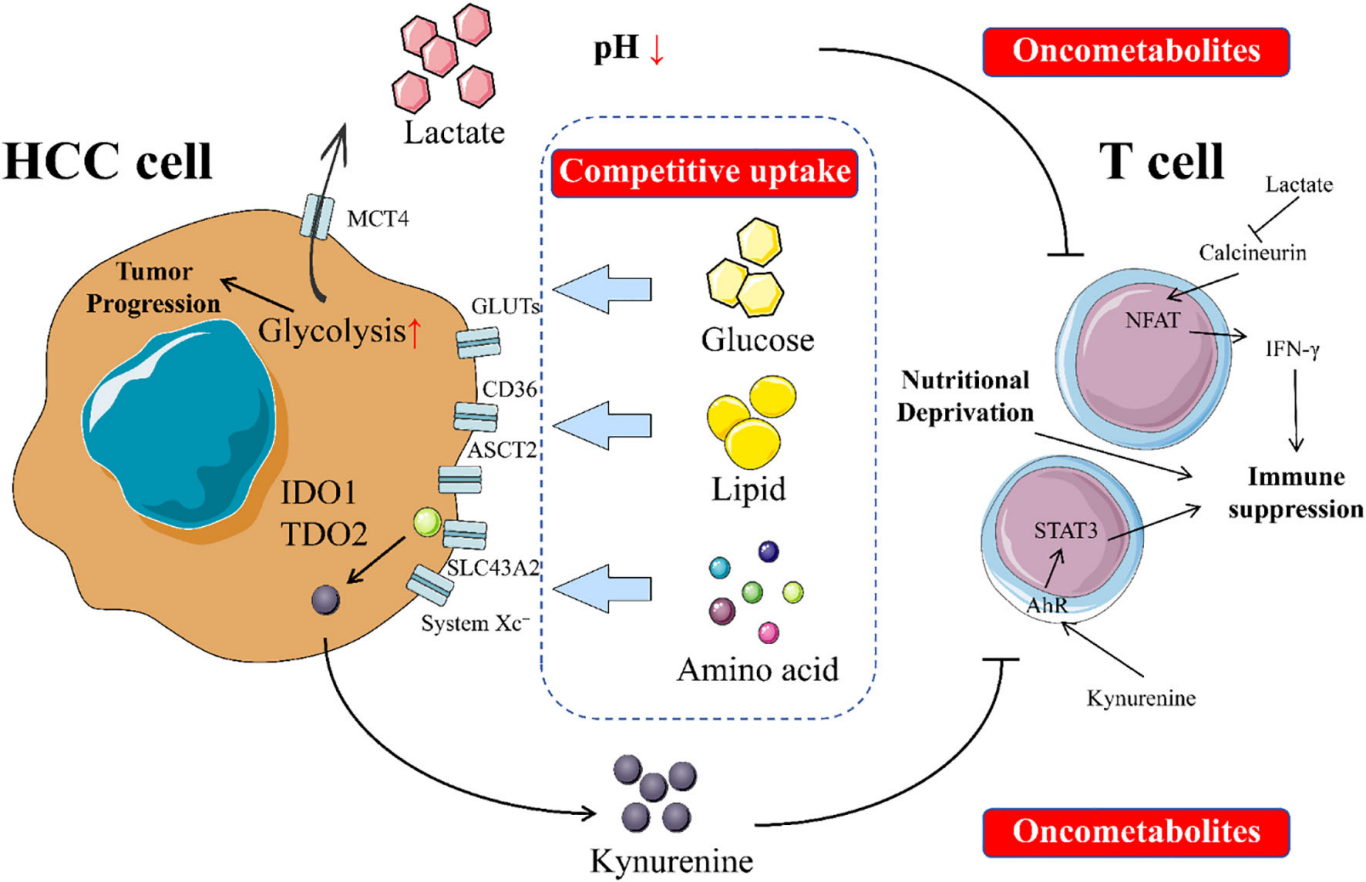
Impacts of metabolic reprogramming on T cells in HCC. In the tumor microenvironment, an intense tug-of-war exists between tumor cells and T cells. Tumor cells compete with T cells for limited nutrients such as glucose, amino acids, and lipids. This competition deprives T cells of the essential nutrients needed for their survival and function, thereby impairing their ability to conduct immune surveillance and kill tumor cells. Additionally, tumor cells secrete oncometabolites that directly harm T cells by disrupting their normal physiological processes, inhibiting their proliferation and activation, and even inducing apoptosis. This interplay not only weakens T cells’ antitumor capabilities but also fosters an environment conducive to tumor growth and spread. The dynamic balance between tumor cells and T cells is crucial for tumor occurrence, development, and immune evasion.

In HCC, cancer cells undergo a series of profound metabolic shifts to meet their immense demands for energy and biosynthetic precursors required for unlimited proliferation. This selfish metabolic reprogramming creates a unique metabolic niche within the TME of HCC, characterized by extreme scarcity of key nutrients and an accumulation of toxic metabolic byproducts (107). Through its distinct metabolic activities, HCC systematically suppresses the antitumor functions of T cells in two main ways: by competitively consuming essential nutrients required by T cells and by releasing inhibitory metabolites that directly “poison” T cells (31, 275, 276). **Figure 1** intuitively illustrates the competition for nutrients between HCC cells and T cells in the tumor microenvironment, as well as the direct damage to T cells caused by the metabolites secreted by tumor cells.

### 4.1 Nutrient competition and depletion: the “starvation” dilemma of T cells

In the microenvironment, rapidly proliferating tumor cells compete with immune cells responsible for immune surveillance, particularly T cells, for the same limited nutrients. With robust metabolic adaptability and reprogramming capabilities, HCC cells often gain an absolute advantage in the competition for survival essentials such as glucose, amino acids, and fatty acids (277).

#### 4.1.1 Glucose depletion

One of the most well-known metabolic traits of HCC cells is their enhanced glycolytic activity, which leads to excessive glucose consumption. However, glucose is the core energy source essential for effector T cells (Teff) to undergo rapid clonal expansion and exert their cytotoxic functions, such as producing key cytokines like interferon-γ (IFN-γ) (278, 279). When T cells are activated and differentiate into effector T cells, their metabolic mode shifts from relying on oxidative phosphorylation (OXPHOS) to being glycolysis-dominant to meet the energy demands for rapid biosynthesis and functional execution (279). Glucose scarcity in the TME caused by excessive consumption by HCC cells, directly results in a severe energy (ATP) shortage for tumor-infiltrating T cells (TILs) (280).

This energy deficit may trigger a cascade of events: impaired mitochondrial function in T cells, inability to sustain normal physiological activities, weakened proliferation capacity, decreased cytokine secretion, and ultimately functional suppression, leading to a state known as “exhaustion” (281). Thus, this glycolysis-based metabolic competition is widely accepted as a key mechanism driving CD8+T-cell exhaustion and resistance to immunotherapy.

#### 4.1.2 Deprivation of key amino acids

Apart from glucose, amino acids are equally vital for the survival, proliferation, and functional differentiation of T cells. HCC cells exhibit strong predatory behavior in this competition.

##### 4.1.2.1 Glutamine

Many HCC cells display a high dependency on glutamine, often referred to as “glutamine addiction.” This excessive consumption significantly reduces the concentration of glutamine in the TME (282). Glutamine, a non-essential amino acid, is primarily transported into cells via the SLC1A5 transporter. SLC1A5 has been shown to be upregulated during T-cell activation, enhancing glutamine uptake. Deprivation of glutamine blocks the proliferation and cytokine production of mouse T cells *in vitro* culture systems (283, 284). Lowering tumor cells consumption of glutamine or increasing the levels of glutamine in the TME through exogenous means may potentially improve the efficacy of related cancer immunotherapies.

##### 4.1.2.2 Arginine

Extracellular arginine is transported across membranes via the y+ system of cationic amino acid transporters (including SLC7A1, SLC7A2, and SLC7A3). Knocking out the expression of SLC7A1 using gene-editing technology reduces T-cell arginine uptake, thereby inhibiting T-cell proliferation. Moreover, arginine deprivation leads to T-cell cycle arrest, loss of the Zeta chain in the T-cell antigen receptor (TCR), and decreased T-cell proliferation and cytokine production (285). In mouse models, exogenous arginine supplementation promotes the generation of central memory T cells, thereby enhancing CD8+T-cell-mediated antitumor activity (286). In addition to protein synthesis, arginine is metabolized into various substances, including nitric oxide, proline, ornithine, creatine, guanidinoacetate, and polyamines. The catabolism of arginine into nitric oxide and its derivative peroxynitrite can inhibit antitumor T-cell responses (287).

##### 4.1.2.3 Methionine

Methionine is an essential sulfur-containing amino acid involved in protein synthesis and the production of S-adenosylmethionine (SAM), which is a key methyl donor for DNA, RNA, and protein methylation modifications (288). Methionine uptake relies on various transporters, such as SLC1A5, SLC7A5, SLC7A6, SLC38A2, and SLC43A2. Notably, SLC7A5 is responsible for leucine uptake that is an essential amino acid for T-cell activation (289). As a result, T cells from SLC7A5-deficient mice exhibit impaired antitumor function due to insufficient uptake of leucine and methionine (290). Interestingly, tumor-infiltrating effector CD8+T cells from humans and mice have lower levels of SLC43A2 expression, leading to reduced intracellular levels of methionine and SAM. This further affects the expression of H3K79me2 and STAT5 and weakening T-cell survival and function (291). Additionally, exogenous supplementation of methionine can significantly restore the antitumor activity of effector CD8+T cells (292).

##### 4.1.2.4 Cystine/cysteine

Cystine enters the cell via the glutamate-cystine antiporter system xCT (encoded by SLC7A11) and is rapidly reduced to cysteine. Although SLC7A11-deficient mouse T cells cannot proliferate *in vitro*, they remain fully activated *in vivo*. Moreover, *in vivo* experiments have shown that the deletion of the SLC7A11 gene in T cells has no clear impact on their antitumor response, while the knockout of the SLC7A11 gene in tumor cells can enhance the efficacy of tumor immunotherapy. This phenomenon may be related to the decreased antioxidant capacity of tumor cells and the increased levels of cystine in the microenvironment (293).

### 4.2 Accumulation of inhibitory metabolites: the “poisonous” environment for T cells

During abnormal and intense metabolic activities, HCC cells actively or passively release large amounts of metabolic byproducts into the TME. These byproducts can severely inhibit T cell function directly or indirectly, collectively creating a “poisonous” environment that leads to T cell dysfunction, exhaustion, or even apoptosis.

#### 4.2.1 Lactate accumulation

Lactate accumulates in large amounts in the TME of HCC. Extremely high concentration of lactate in the TME creates a reverse concentration gradient. This hinders the efflux of lactate produced by T-cell metabolism and drives lactate from the TME into T cells via monocarboxylate transporters such as MCT1, leading to intracellular lactate accumulation and acidification (60, 294). High intracellular lactate concentration competitively inhibits the activity of lactate dehydrogenase (LDH), disrupting the NAD+/NADH redox balance in the cytoplasm and, in turn, negatively feedback-inhibiting key glycolytic rate-limiting enzymes such as phosphofructokinase-1 (PFK-1) (295). Ultimately, this plunges T cells into an “energy crisis,” preventing them from maintaining efficient effector functions. Studies have clearly shown that the acidic environment of the TME (low pH) directly impairs the conformation and enzymatic activity of calcineurin. Calcineurin, a phosphatase whose activity is inhibited, cannot effectively catalyze the dephosphorylation of NFAT proteins (296). This “locks” NFAT in the cytoplasm, preventing it from entering the nucleus to bind to the promoters of target genes, thereby severely weakening or even completely blocking the transcription and secretion of cytokines such as IFN-γ (297). Thus, the acidosis caused by lactate essentially cuts off the “command system” for T-cell-initiated immune responses. Moreover, Tumor-derived lactic acid modulates dendritic cell differentiation, inducing an altered phenotype that reduces IL-12 secretion and contributes to tumor immune escape (212).

#### 4.2.2 Kynurenine

Tryptophan is an amino acid essential for T-cell proliferation and function (298). However, in the TME, tumor cells, stromal cells, or myeloid-derived suppressor cells (MDSCs) highly express indoleamine 2,3-dioxygenase (IDO1) or tryptophan 2,3-dioxygenase (TDO) (299). These enzymes catalyze the catabolism of tryptophan via the kynurenine pathway. This process exerts a dual inhibitory effect: 1) it depletes tryptophan in the TME, causing “nutritional starvation” of T cells and triggering their stagnation and anergy; and 2) its metabolite kynurenine and downstream derivatives, as endogenous ligands of the aryl hydrocarbon receptor (AHR), can be taken up by T cells and activate the AHR signaling pathway within them (211, 300). AHR activation has been shown to inhibit the function of effector T cells and promote the differentiation and proliferation of regulatory T cells (Tregs) with immunosuppressive capabilities, thereby further deepening immune suppression (301).

#### 4.2.3 Lipid overload

Liver is the center of lipid metabolism, and thus HCC is often accompanied by significant lipid metabolic disorders. This finding is not only reflected within tumor cells but also throughout the entire TME, leading to the accumulation of large amounts of lipid components such as free fatty acids (FFAs), cholesterol, and their derivatives (302). This lipid-rich environment is a “double-edged sword” for infiltrating T cells, ultimately leading to functional impairment (303).

Excessive cholesterol uptake and ER stress: Tumor-infiltrating CD8+T cells take up large amounts of lipids from the TME via scavenger receptors (such as CD36) on their surface, attempting to use them as alternative energy sources for fatty acid oxidation (FAO) (304). However, the influx of excessive lipids, particularly free cholesterol, exceeds the cells’ normal metabolic and storage capacities, accumulating within the cells, especially in the endoplasmic reticulum (ER), and causing severe “lipotoxicity”. The accumulation of free cholesterol disrupts the integrity and fluidity of the ER membrane, interfering with proper protein folding and triggering “ER stress” and the broader “unfolded protein response” (UPR) (305). Persistent, unresolved ER stress is a harbinger of cellular dysfunction and apoptosis.

ER stress-driven T-cell exhaustion: ER stress is not a passive damage response. It actively activates a series of downstream signaling pathways that directly push T cells into an “exhaustion” state. Studies have shown that key molecules in ER stress, such as the spliced active form of X-box-binding protein 1 (XBP1s), can act as transcription factors to directly bind to and activate the promoters of genes encoding immune checkpoint molecules related to T-cell exhaustion, such as PD-1, T-cell immunoglobulin and mucin-domain-3 (TIM-3), and lymphocyte-activation gene-3 (LAG-3) (305, 306). The upregulation of these checkpoint molecules makes T cells abnormally sensitive to inhibitory signals from tumor cells, ultimately inducing their dysfunction, proliferative arrest, and apoptosis.

Unlike effector T cells that primarily rely on glycolysis, memory T cells preferentially utilize FAO to sustain their long-term survival and function. This metabolic dichotomy suggests that enhancing FAO through activation of enzymes like PPARα or CPT1A can improve memory T cell function and anti-tumor immunity (307–310). Thus, therapeutic strategies targeting lipid metabolism require precise modulation to effectively suppress tumor metabolism while preserving the critical functions of memory T cells.

**Figure 2:**
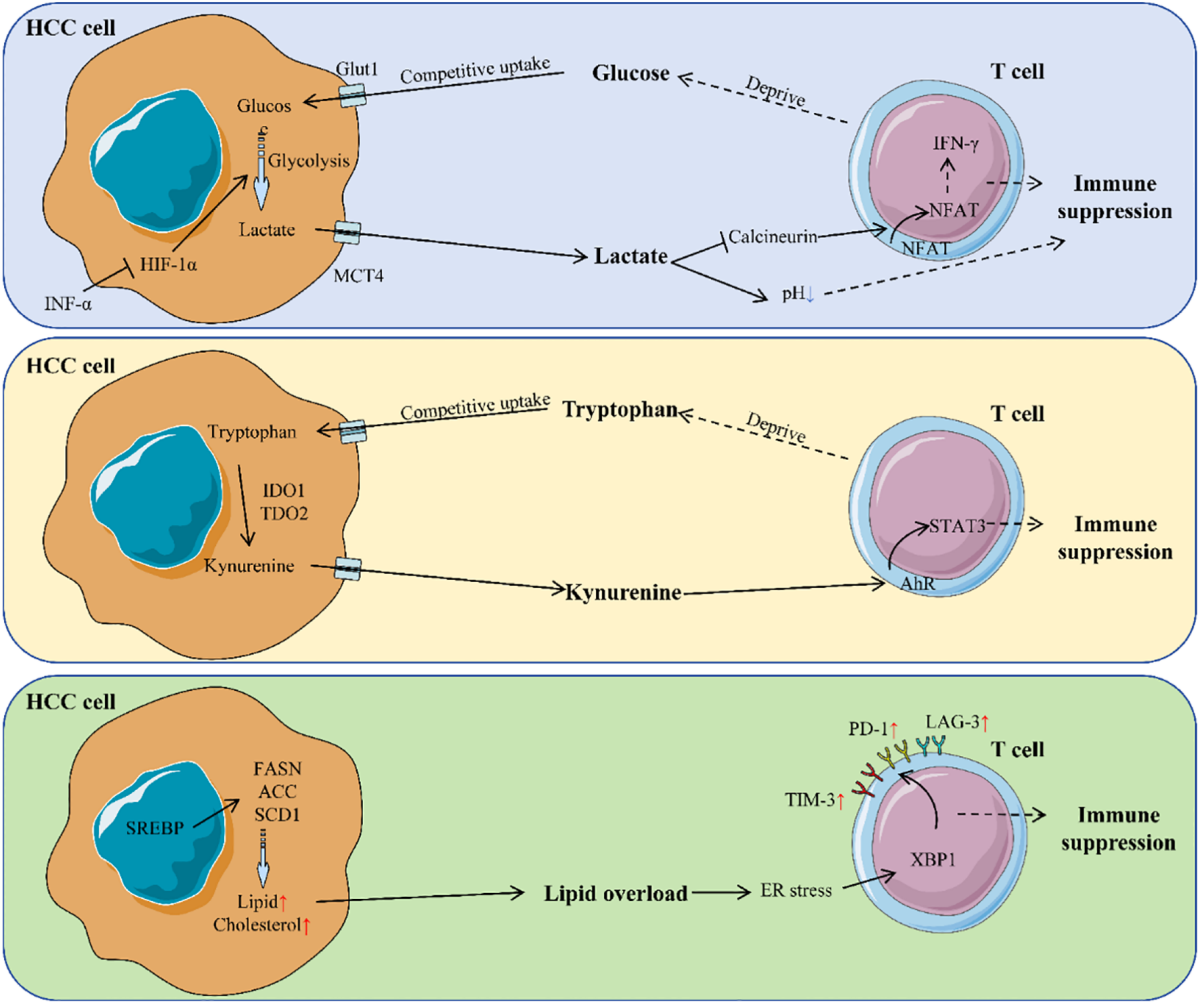
Mechanism of metabolic reprogramming regulating T cells in HCC. HCC cells reprogram metabolism to create an immunosuppressive TME. They consume glucose, produce lactate, and disrupt tryptophan metabolism. Lactate acidifies the TME, impairing T cell activity by inhibiting calcineurin activity in T cells, preventing NFAT from entering the nucleus and thus suppressing the secretion of cytokines like IFN-γ. Tryptophan breakdown generates kynurenine, which activates the AHR pathway in T cells, further suppressing their function. Lipid overload in the TME causes ER stress in T cells, leading to upregulation of immune checkpoint molecules and T cell exhaustion. These metabolic changes collectively inhibit T cell function, promoting immune evasion.

**Figure 2** comprehensively illustrates the mechanisms by which metabolic reprogramming regulates T cell functions through multiple pathways, including the effects of glucose metabolism, lactate production, and tryptophan metabolism on T cells. **Table 2** details the specific effects of changes in various metabolites on T cells and their underlying molecular mechanisms.

**Table 2:** Mechanisms of metabolite effects on T cells.

Metabolite alteration	Impact on T cells	Molecular mechanism	Refs
Glucose ↓	Proliferation ↓, IFN-γ secretion ↓	Energy crisis → Mitochondrial dysfunction → Exhaustion	(268, 269)
Glutamine ↓	Activation blocked	SLC1A5-dependent uptake essential for T cell activation	(271, 272)
Arginine ↓	Cell cycle arrest, TCR signaling ↓	SLC7A1-mediated arginine uptake ↓ → Zeta chain loss	(273, 274)
Lactate ↑	Cytotoxicity ↓, Immune response paralysis	TME acidification → Calcineurin inhibition → Blocked NFAT nuclear translocation	(58, 285)
Kynurenine ↑	Treg differentiation ↑, Teff function ↓	AHR activation → STAT3 phosphorylation → Immunosuppressive gene expression	(289, 290)
Cholesterol ↑	CD8+ T cell exhaustion	Lipid overload → ER stress → XBP1s-mediated PD-1/TIM-3 upregulation	(295, 296)

### 4.3 The impact of gut microbiota on metabolic reprogramming and T-cell responses

The gut microbiota contributes to the pathogenesis of HCC through metabolic regulation and immune modulation. As a complex microbial ecosystem within the human body, its metabolic activities continuously influence host physiology and disease states through the production of bioactive molecules. Recent studies have revealed that gut microbiota and their metabolites play critical roles in HCC initiation and progression, metabolic reprogramming, and shaping the tumor immune microenvironment (311–313). Specific microbial metabolites, such as short-chain fatty acids (SCFAs) and secondary bile acids, directly influence HCC metabolic adaptability and T-cell responses by modulating hepatic metabolic status and immune cell functions (314–316).

#### 4.3.1 Regulation of hepatic metabolism by microbial metabolites

Gut microbial metabolites are transported to the liver via the portal vein, directly regulating the physiological functions of hepatocytes and hepatic immune cells (317). SCFAs, the main products of dietary fiber fermentation by gut bacteria, modulate hepatocyte metabolism through signaling pathways mediated by G protein-coupled receptors (GPR41, GPR43) (318). They alleviate hepatic steatosis by promoting fatty acid oxidation and inhibiting lipid accumulation (319, 320). Under conditions of gut dysbiosis, decreased SCFA levels may exacerbate hepatic metabolic disorders, thereby promoting HCC progression (314). Secondary bile acids, derived from microbial transformation of primary bile acids, regulate hepatic metabolic homeostasis through the farnesoid X receptor (FXR) and Takeda G protein-coupled receptor 5 (TGR5) (321, 322). Studies have demonstrated that abnormal accumulation of secondary bile acids is significantly associated with hepatic steatosis, inflammatory responses, and HCC development (321, 323). Dysbiosis-induced excessive accumulation of secondary bile acids can activate pro-inflammatory signaling pathways, driving hepatic inflammation and fibrosis, thereby creating a favorable environment for HCC initiation (324).

#### 4.3.2 Modulation of T-cell responses by microbial metabolites

Gut microbial metabolites also regulate T-cell function in the liver through immunometabolic reprogramming mechanisms. SCFAs can enhance the differentiation and function of Tregs by influencing immunocyte metabolism, thereby maintaining immune tolerance (325). However, in HCC patients, reduced SCFAs due to dysbiosis may impair Treg function, exacerbate hepatic inflammation, and foster an immunosuppressive tumor microenvironment (326). Secondary bile acids modulate immune cell activity via the TGR5 receptor. Normal activation of TGR5 promotes anti-inflammatory cytokine expression and suppresses excessive inflammatory responses (327, 328). Nevertheless, abnormal accumulation of secondary bile acids caused by dysbiosis may impair TGR5 signaling, weaken anti-inflammatory protective mechanisms, and ultimately promote HCC progression (323, 324).

## 5 Metabolic-immune targeted combination therapy for HCC

Metabolic reprogramming in HCC can suppress the antitumor activity of T cells. Based on an in-depth understanding of this mechanism, researchers are actively exploring strategies to combine metabolic pathway inhibitors with immune checkpoint inhibitors (ICIs) to achieve a synergistic effect in HCC treatment (31).

### 5.1 Combination strategies targeting glucose metabolism

It has been reported that IFNα can ameliorate the glucose-deprived state within the TME by reprogramming glucose metabolism in the HCC TME, thereby enhancing antitumor immune activity. Mechanistically, IFNα suppresses HIF1α signaling by inhibiting FBJ osteosarcoma oncogene B (FosB) transcription in HCC cells, leading to reduced glucose consumption capacity in tumor cells and consequently establishing a high-glucose microenvironment. This glucose-enriched milieu promotes transcription of the costimulatory molecule Cd27 in infiltrating CD8+ T cells via the mTOR-FOXM1 signaling pathway, releasing T-cell cytotoxic potential and thereby potentiating the PD-1 blockade-induced immune response (329).

### 5.2 Combination strategies targeting glutamine metabolism

Glutaminase (GLS) inhibitors, such as CB-839/Telaglenastat, are among the most extensively studied drugs in this field. Multiple preclinical studies have demonstrated that the combination of CB-839 with anti-PD-1/PD-L1 antibodies exhibits significant synergistic antitumor effects in various tumor models, including non-HCC models. The mechanism primarily involves inhibiting glutamine catabolism, thereby alleviating the nutrient deprivation of T cells in the TME and promoting the activation and tumor infiltration of effector T cells (330–332).

### 5.3 Combination strategies targeting lipid synthesis

The combination of fatty acid synthase (FASN) inhibitors with ICIs has also shown therapeutic potential in HCC models. A study demonstrated that in an orthotopic HCC mouse model, FASN inhibition enhanced the stability of major histocompatibility complex class I (MHC-I) molecules and synergized with anti-PD-L1 blockade to significantly inhibit tumor growth (333). Nevertheless, in another study based on the sgPTEN/c-Met genetically engineered mouse model, no synergistic effect was observed between the FASN inhibitor TVB3664 and the anti-PD-L1 antibody. This suggests that the efficacy of combination therapy may depend on specific tumor genetic backgrounds and microenvironmental characteristics (334). However, the latest detailed preclinical data on the combination of FASN inhibitors with ICIs for HCC treatment, especially studies including systematic immune cell profiling, are also relatively scarce (334, 335).

### 5.4 Metabolic plasticity, drug resistance, and dynamic metabolic inhibition strategies

Metabolic plasticity is a key adaptive characteristic of cancer cells that enables them to reprogram metabolic pathways to sustain survival and proliferation under metabolic-targeted therapies. In combined therapies for HCC, although metabolic–immune targeting strategies may initially demonstrate considerable anti-tumor efficacy, the metabolic plasticity of cancer cells often leads to acquired resistance. For instance, when GLS inhibitors (e.g., CB-839) are combined with immune checkpoint inhibitors, tumor cells may activate alternative metabolic routes, such as the glutamine synthesis pathway, to circumvent the metabolic stress induced by GLS inhibition (336, 337). Such metabolic reprogramming allows cancer cells to persist under drug pressure, thereby compromising treatment outcomes. This dynamic adaptation of metabolic pathways not only diminishes the efficacy of single-target inhibitors but may also impair immune cell function, as immune activity relies on specific metabolic states.

To overcome the metabolic plasticity of cancer cells, future therapeutic approaches may require the implementation of dynamic or sequential metabolic inhibition strategies. The core concept involves co-targeting multiple metabolic pathways to disrupt the adaptive capacity of cancer cells, thereby enhancing treatment efficacy and reducing the incidence of resistance. For example, initial inhibition of glutamine metabolism using a GLS inhibitor could be followed by combination with a PPP pathway inhibitor (e.g., Oxythiamine) to further restrict metabolic flexibility. Such sequential targeting effectively depletes metabolic resources, impeding cancer cell survival through singular pathway reprogramming. In the context of lipid metabolism, combined administration of FASN inhibitors and HMGCR inhibitors (e.g., statins) may be considered. FASN inhibition reduces lipid synthesis, while statins suppress cholesterol production, thereby dually restricting lipid availability. This combined suppression not only directly affects the metabolic state of cancer cells but may also enhance immune cell function by modulating lipid levels in the tumor microenvironment. Additionally, dynamic metabolic inhibition strategies can be integrated with immunotherapy. For instance, concurrent use of metabolic inhibitors and immune checkpoint blockers may improve immune-mediated recognition and elimination of cancer cells. Such comprehensive therapeutic approaches not only directly suppress tumor metabolism but may also amplify treatment efficacy by remodeling the immune microenvironment.

## 6 Conclusion

This review provides a comprehensive overview of the metabolic reprogramming in HCC and its profound impact on T cell-mediated antitumor immunity. We have detailed the various metabolic alterations in HCC cells, including enhanced glycolysis (the Warburg effect), lipid metabolism changes, and amino acid metabolism reprogramming, which collectively create an immunosuppressive TME. These metabolic shifts lead to nutrient depletion and the accumulation of inhibitory metabolites, such as lactate and kynurenine, which directly impair T cell function and promote T cell exhaustion.

The innovation of this review lies in its comprehensive analysis of the metabolic-immune interactions in HCC, highlighting the dual role of metabolic reprogramming in fueling tumor growth and suppressing antitumor immunity. By elucidating the mechanisms through which HCC cells outcompete T cells for essential nutrients and produce inhibitory metabolites, this review provides a novel perspective on the therapeutic bottleneck of current immunotherapies. The practicality of this work is evident in its exploration of potential combination therapies targeting metabolic pathways alongside ICIs, offering new strategies to enhance treatment efficacy and overcome resistance in HCC.

This review has several limitations that should be considered. A primary constraint stems from its reliance on published literature, which introduces potential publication bias due to the omission of unpublished or negative results. Moreover, the high degree of heterogeneity in HCC and the dynamic complexity of the TME complicate the extrapolation of findings, as metabolic profiles and their impact on immunity may differ significantly among patients. Although we highlight promising metabolic-immune combination strategies, most supporting evidence comes from preclinical studies. Clinical data remain sparse, with limited sample sizes and follow-up, thereby necessitating further validation of the long-term efficacy and safety of these approaches. Future studies are warranted to address these gaps through expanded clinical trials and mechanistic validation in diverse experimental models.

Future research should focus on several interconnected areas to advance the understanding and treatment of HCC. A deeper exploration of the metabolic adaptations of immune cells within the tumor microenvironment and their dynamic interplay with cancer cell metabolism will help identify novel therapeutic targets. Complementing this, comprehensive and patient-specific metabolic profiling of HCC tumors is essential to fully elucidate the relationship between metabolic reprogramming and immune evasion. The integration of advanced technologies such as spatial metabolomics will be critical to decipher the spatiotemporal heterogeneity of metabolic pathways and provide a foundation for precision medicine. Furthermore, the role of the gut microbiota and its metabolites in the metabolic-immune network of HCC warrants further mechanistic investigation for potential clinical intervention. On the translational front, efforts should prioritize validating the efficacy of metabolism-immunity combination therapies in larger clinical cohorts and developing novel biomarkers to predict patient response, thereby enabling personalized treatment strategies. Finally, exploring additional metabolic pathways and their immunomodulatory effects, coupled with fostering interdisciplinary collaboration to leverage new materials and technologies, will be vital to uncover new therapeutic opportunities and accelerate the translation of basic findings into effective clinical applications for HCC.

## Glossary

HCC: Hepatocellular carcinoma
TME: Tumor microenvironment
ICI: Immune checkpoint inhibitor
GLUT1: Glucose transporter 1
HK2: Hexokinase 2
PKM2: Pyruvate kinase M2 isoform
LDHA: Lactate dehydrogenase A
HIF-1α: Hypoxia-inducible factor 1α
PFK1: Phosphofructokinase 1
AMPK: AMP-activated protein kinase
PPP: Pentose phosphate pathway
G6PD: Glucose-6-phosphate dehydrogenase
TKT: Transketolase
FBP1: Fructose-1,6-bisphosphatase 1
PCK1: Phosphoenolpyruvate carboxykinase
IDH2: Isocitrate dehydrogenase 2
ME1: Malic enzyme 1
ACLY: ATP citrate lyase
ACC: Acetyl-CoA carboxylase
FASN: Fatty acid synthase
SCD1: Stearoyl-CoA desaturase 1
ACSL4: Acyl-CoA synthase long-chain family member 4
SREBP1: Sterol regulatory element-binding protein 1
LXR: Liver X receptor
FAO: Fatty acid oxidation
CPT1: Carnitine palmitoyltransferase 1
MCAD: Medium-chain acyl-CoA dehydrogenase
LCAD: Long-chain acyl-CoA dehydrogenase
MAGL: Monoglyceride lipase
ATGL: Adipose triglyceride lipase
HMGCR: HMG-CoA reductase
PPARα: Peroxisome proliferator-activated receptor α
CYP4: Cytochrome P450 family 4
GLS: Glutaminase
SLC1A5: Solute carrier family 1 member 5
SLC7A5: Solute carrier family 7 member 5
GOT1: Glutamic oxaloacetic transaminase 1
CPS1: Carbamoyl phosphate synthetase 1
ASS1: Argininosuccinate synthase 1
ARG1: Arginase-1
OTC: Ornithine transcarbamylase
IDO1: Indoleamine 2,3-dioxygenase 1
TDO2: Tryptophan 2,3-dioxygenase 2
MCT: Monocarboxylate transporter
AHR: Aryl hydrocarbon receptor
TIM-3: T-cell immunoglobulin and mucin-domain-3
LAG-3: Lymphocyte-activation gene-3
SCFAs: Short-chain fatty acids
FXR: Farnesoid X receptor
TGR5: Takeda G protein-coupled receptor 5
IFN-γ: Interferon-γ
IL-12: Interleukin-12
PD-1: Programmed death receptor-1
PD-L1: Programmed Death-Ligand 1
